# Barriers to accessing cervical cancer screening among HIV positive women in Kgatleng district, Botswana: A qualitative study

**DOI:** 10.1371/journal.pone.0205425

**Published:** 2018-10-24

**Authors:** Tjedza G. Matenge, Bob Mash

**Affiliations:** Division of Family Medicine and Primary Care, Stellenbosch University, Cape Town, South Africa; Makerere University School of Public Health, UGANDA

## Abstract

**Background:**

Low and middle-income countries have a greater share of the cervical cancer burden, but lower screening coverage, compared to high-income countries. Moreover, screening uptake and disease outcomes are generally worse in rural areas as well as in the HIV positive population. Efforts directed at increasing the screening rates are important in order to decrease cancer-related morbidity and mortality. This study aimed to explore the barriers to women with HIV accessing cervical cancer screening in Kgatleng district, Botswana.

**Methods:**

A phenomenological qualitative study utilising semi-structured interviews with fourteen HIV positive women, selected by purposive sampling. The interviews were transcribed verbatim and the 5-steps of the framework method, assisted by Atlas-ti software, was used for qualitative data analysis.

**Results:**

Contextual factors included distance, public transport issues and work commitments. Health system factors highlighted unavailability of results, inconsistent appointment systems, long queues and equipment shortages and poor patient-centred communication skills, particularly skills in explanation and planning. Patient factors identified were lack of knowledge of cervical cancer, benefits of screening, effectiveness of treatment, as well as personal fears and misconceptions.

**Conclusion:**

Cervical cancer screening was poorly accessed due to a weak primary care system, insufficient health promotion and information as well as poor communication skills. These issues could be partly addressed by considering alternative technology and one-stop models of testing and treating.

## Introduction

Cervical cancer is a global public health issue, affecting not only women, but also their families, communities and social institutions.[1] It is the second leading cause of cancer-related mortality among women globally, although it is also one of the most preventable cancers due to its slow progression, identifiable cytological precursors and effective treatment.[1,2] Even though the disease is preventable, more than half a million women worldwide develop invasive cervical cancer each year.[3] Low and middle income countries bear a disproportionate share (83%) of the global cervical cancer burden, but only manage an average screening coverage of 19%, compared to 63% in high income countries.[2] Screening uptake and disease outcomes are generally worse in rural areas compared with urban areas because the population is substantially poorer and access to health services is more difficult.[3] The burden of disease from cervical cancer also falls more on older women who are post-childbearing and who are important to both family stability and the broader economy.[4]

In African countries, such as Malawi, cervical cancer is the most prevalent form of cancer among women with an 80% mortality rate.[5] Efforts directed at increasing the cancer screening rates are important in order to decrease the number of cancer deaths.[6] In the UK, the cervical screening programme has been successful in securing participation of a high proportion of targeted women and has seen a fall in mortality rates.[7] A sharp decline in cervical cancer incidence in the USA over the past 50 years has also been attributed to the increase in screening using the Papanicolaou (pap) smear test, which is relatively cheap and widely available.[8,9] Visual inspection with acetic acid and cervicography (VIAC), followed by immediate cryotherapy or a loop electrical excision procedure (LEEP), is an alternative approach in resource limited areas.[10]

The prevalence of HIV in Botswana is estimated to be 23% in the general adult population, making Botswana’s HIV prevalence the second highest in the world.[11] Moreover, there is a positive association between HIV/AIDS and cervical cancer with the cancer being more common and progressing faster.[12] Cervical cancer is caused by Human Papilloma Virus strains (HPV) 16, 18 and 31. Both HPV and HIV are largely transmitted through sexual intercourse. Moreover, cervical cancer is a stage IV disease in the World Health Organization’s HIV clinical staging guidelines and is an AIDS defining condition.[13]

Extensive research on women’s knowledge of and access to cervical cancer screening has been conducted in countries such as USA, UK and Australia.[14] Access to cervical cancer screening has been found to be disproportionate across socio-economic groups and higher rates of invasive cervical cancer exist among women of lower income and educational levels.[9] Access to care is affected by a complex set of interacting factors that may present challenges for individual women such as the social environment, health behaviours, occupational factors, biological variables, cultural beliefs and values.[9]

Few studies have been conducted in an African context. Utilisation of cervical cancer screening services is low, especially in lower socio-economic communities and rural areas.[15,16,17,18] There is a need to understand women’s motivation and factors affecting utilisation of services.[19] This study will contribute to a better understanding of the barriers to cervical cancer screening among women with HIV in Botswana. The aim of the study was to explore the barriers for women with HIV from accessing cervical cancer screening in Kgatleng district, Botswana. Specific areas to explore included their ideas and beliefs regarding cervical cancer screening as well as their experience of the health services and quality of care.

## Methods

### Study design

This was a phenomenological qualitative study utilising key informant semi-structured interviews.[20]

### Setting

The study was conducted in Oodi village in the Kgatleng district of Botswana. This rural village is about 20 km from the capital city of Gaborone. Oodi clinic is the only clinic and offers a broad range of chronic, acute, emergency, maternal, preventative and medico-legal services.

The staff at the HIV clinic included a medical officer, registered nurses, midwives, a psychiatric nurse, ARV prescriber nurses, ARV dispenser nurses, a pharmacy technician and other support staff. Women were referred to the clinic from two other health posts and a smaller clinic. Cervical cancer screening was carried out by the midwives and nurse practitioners in the general clinic. Women either presented to them voluntarily, were referred because of symptoms or were motivated to attend during clinic visits. Women at the HIV clinic were meant to be regularly reminded to have a cervical smear. Women with abnormal cervical smears were referred to the national tertiary Princess Marina Hospital in Gaborone.

The researcher was a female medical officer working in the Greater Gaborone District, but not at Oodi clinic and she had no prior relationship with the study participants. She was of the same broad *Setswana* culture as most of the residents and spoke the local language, but was not of the same sub-culture as the Bakgatla in Oodi village.

### Study population

The study population was HIV positive females, 21 years of age and above, who attended the HIV clinic, irrespective of whether they had started highly active anti-retroviral therapy (HAART) or not and irrespective of where they resided.

### Study participants

Initially six women were interviewed in order to explore the range of their responses and experiences using purposive sampling, based on the following criteria:

- Half the women should not have accessed cervical screening in the past 15 years
- Half the women should have accessed cervical screening in the past 15 years
- Equal numbers of women from three age categories (21–29 years, 30–39 years, 40 years or older).

Further interviews were conducted to explore all the issues and until no new themes were recognised, at which point there were 14 interviews. The researcher attended the study site on one day a week and usually interviewed two people at each visit. The nursing staff helped to identify suitable participants in the clinic on that day, who met the criteria and were likely to have an opinion on the topic that they were willing to share. None of the women selected refused to participate.

### Data collection

Interviews were conducted (February-June 2016) in the *Setswana* language by the researcher who was trained in interviewing skills at Stellenbosch University. Women were interviewed in a separate room from the HIV clinic and cervical screening area in order to be more neutral and to make the interviewees more comfortable. The individual interviewees were welcomed and basic demographic information obtained. The interviews were audio recorded and ranged from 28–47 minutes. Participants were encouraged to voice their opinions spontaneously and openly without having to wait for specific questions. An interview guide (S1 File) that had all the important broad issues in the form of open-ended questions was used to ensure the topic was fully explored.

### Data analysis

The interviews were transcribed verbatim. Before analysing the data the transcripts were checked for accuracy and any mistakes corrected against the original recording.[21] Analysis was performed in *Setswana* and only important quotes were translated to English for reporting purposes. The 5-steps of the framework method for qualitative data analysis with the help of Atlas-ti software was used:[21]

- Familiarisation: This started in the field while collecting data. Once the data had been collected, the researcher familiarised herself with the data as a whole by reading the transcripts and listening to the tapes. Potential codes as well as her own reaction to the data were noted together with any issues regarding the quality of the data.
- Development of the thematic index: A list of codes was created based on the familiarisation process above and organised into categories. Codes were developed from the data (an inductive process), and the categories reflected the study objectives.
- Indexing: Here the codes in the thematic index were systemically applied to all the qualitative data.
- Charting: Data was re-arranged in a series of charts that brought all the data with the same code together in one place from all the data sources.
- Interpretation: Each chart was read and interpreted. It was now possible to establish the range and nature of the phenomenon of interest. Deviant cases were also given attention as they helped to further understand the phenomenon. Any associations or relationships between themes were interpreted.

The qualitative data analysis process was supervised by the second author who was an experienced qualitative researcher. Supervision focused on construction of the thematic index and interpretation of the charts, while indexing was done by the principal researcher.

Themes were categorised into contextual issues, health system related issues and patient related issues. Contextual issues related to factors external to the patient and health system such as occupational or transport related issues. Health system issues included organisational processes and consultation skills such as health education, explanation and planning. Patient related issues referred to the patient’s perspective on cervical cancer and prevention. The participants are labelled as 1, 2, 3 up to 14 in the quotations provided.

## Ethical considerations

Ethical approval was obtained from the University of Stellenbosch Health Research Ethics Committee (S15/01/006) as well as from the Ministry of Health of Botswana (HPDME 13/18/1 X (2).

## Results

A total of 14 women were interviewed and their characteristics are described in Table 1. Overall, eight women had accessed screening and six had not, three were aged 21–29 years, five were aged 30–39 years and six were aged 40 years or more. All were from Oodi village, apart from one woman who was a farm worker from Kgaphamadi Gaborone North farms.

**Table 1:** Characteristics of participants.

Participant	Age in years	Parity	Year of last cervical smear screening	Highest level of education attended	Occupation
1	49	4	1998	High school	Cleaner
2	29	1	2014*	High school	Unemployed
3	48	2	Never done	Primary school	Domestic worker
4	34	2	2015	High school	Cleaner
5	32	3	2013	High school	Hairdresser
6	37	4	Never done	High school	Unemployed
7	41	2	2012	High school	Domestic worker
8	40	3	Never done	High school	Cleaner
9	39	6	2012	Primary school	Farm worker
10	29	1	Never done	High school	Unemployed
11	43	9	2014	Primary school	Unemployed
12	42	2	2009; 2014	High school	Cleaner
13	29	2	2013	Diploma in Accounting	Accountant
14	32	3	2013	High school	Unemployed

* Had visual inspection with acetic acid

### Contextual issues

The distance from the clinic and the unpredictability of public transport limited access. Women that were employed complained that it was difficult to take time off work as they would have deductions from their pay if they were absent to attend the clinic. Going to the clinic depended on how important they perceived the clinic visit to be, as women were committed to attend the HIV clinic in order to obtain anti-retroviral medication, but did not give the same level of importance to attending for a cervical smear:

*“I have never done the pap test because of the kind of job I have. If you constantly ask to be excused from work…aah… They say you shouldn’t ask for permission daily but I told them my life comes first and I would rather lose the job. I told them my life comes first when I am going to collect my ARV’s nobody can stand in my way … I told them if the issue is money I would rather have unpaid days because money can never equate to my life.” (Participant 1)*.

### Health system related issues

Many participants never received their result or only obtained it months or years later. Some even ended up having to repeat the test, with no explanation offered as to why. There was deep concern expressed by many that the longer the results took to come out, the more the cancer could be spreading:

*“The ones that delayed are those that I got last year because I tested in 2015 and only got the result beginning of last year. It means they took a year*.*” (Participant 4)*

*“It did not sit well with me because I did not know what my cancer status is, because the longer it takes to get them [results], the more the cancer could be progressing and spreading, if it is there*.*” (Participant 14)*

The problems encountered with getting the results led some women to repeat the cervical smear in multiple places in order to increase the chances of getting the results. Furthermore, the delays in getting the results seemed to be a problem not only at the Oodi clinic, but at other health facilities too. Women were even more dissatisfied when they were told to come for the results after a specified period, only to find that they were not available. Women felt that they should not have to come multiple times to obtain their results and that they were owed an explanation of why the results were not available:

*“Once did pap smear and when I checked results I was told to come after 6 months, so I was told they are unavailable so I ended up giving up on them and going to redo. Was disappointed because I took a very long time still waiting for results*.*” (Participant 2)*

Women also reported frustrations with the local clinics and even the district hospital for not having all the equipment to perform the cervical smear. For first time users of the service this might demotivate them from trying again, while others might bypass the service and present themselves at the referral hospital. Others were told their results were abnormal, but could not repeat the test due to equipment shortages:

*“Yes, madam and I got the first results but they said they have seen something unusual so they said I have to come again. I came back and they said testing tools are unavailable. I kept on coming until I became a bit discouraged*.*” (Participant 9)*

*“Sometimes when you come they say some things are not here so we have to go check at a bigger hospital like Marina [referral hospital]*.*” (Participant 6)*

Some women were discouraged by having to make an appointment for the cervical smear. Clinic procedures were inconsistent with some operating without appointments and others requiring them. Women could be turned away if they did not have an appointment, even though they wanted a cervical smear:

*“I feel it discourages them a lot, even this issue that you have to book before you do pap smear discourages…There is where you just get in the queue but in some where you have to book*.*” (Participant 4)*

Nevertheless, other women complained of long queues and waiting times, which also discouraged them from accessing cervical smears:

*“Maybe I can say the clinic is far, and also long queues and also sometimes they say they don’t have tools*.*” (Participant 7)*

One respondent reported that there was a shortage of nurses and therefore she was unable to predict on which days the service would be available:

*“I went there and they said there was shortage of nurses*.*” (Participant 3)*

All of the difficulties encountered led to a loss of confidence in the clinic’s ability to provide the service. Some suggested that the service offering direct visual inspection with acetic acid (VIA) and immediate treatment was preferable or that women should try and obtain both VIA and a cervical smear:

*“I can advise them to do both [VIA and cervical smear] so that when still waiting for results so that if they have cancer they can be treated*.*” (Participant 2)*

Healthcare workers might instruct women to have a cervical smear without explaining why this was necessary. Women would then be less motivated to make the effort. Similarly, the healthcare workers might not fully explain the significance of an abnormal result or speak in a language that the woman could not understand:

*“I did not really understand, if only I had tested again I would know what my results are but I don’t know my results*.*” (Participant 9)*

Being told to then repeat the test without a full understanding as to why it was important and negative attitudes from clinic staff could further explain a loss to follow up:

*“Nurses don’t treat us well. When you get there, they start saying a lot of different things*.*” (Participant 11)*

### Patient related issues

There was a general lack of knowledge about screening and cervical cancer in general and this could have affected attendance:

*“Maybe you should explain*, *pap smear is what exactly?” (Participant 8)*

Lack of knowledge on the risk factors for cervical cancer could further affect the way HIV positive women viewed the importance of going for a cervical smear. Many did not associate the disease with sexual intercourse. They believed that penis size during sexual intercourse and potential trauma was a risk factor. Others misinterpreted the giving of the HPV vaccine to young girls prior to sexual debut as an indication that they were at immediate risk of cervical cancer:

*“I have never taken time to think about the issue because these days I do not even understand as you can find that the least expected people end up having cervical cancer…more so that these days even babies get vaccinated against cervical cancer*.*” (Participant 4)*

*“I have heard that even small children can have cervical cancer even though they have never had sex*.*” (Participant 12)*.

*“People come in different sizes and you may use a condom but if you encounter a big penis size and get some injuries internally then I don’t think that can prevent the cervix from getting damaged or infected or something*.*” (Participant 13)*

The lack of knowledge on the recommended frequency of testing was widespread and many believed that only one test was necessary. Others thought that you only needed to be tested if you had symptoms such as a vaginal discharge:

*“I had thought that if they say that it is okay I don’t have cancer then I do not need to check again*.*” (Participant 1)*

*“When you find whitish discharge on the lining… that comes from the cervix, then you have to go for a pap test coz then you don’t know if the cervix is ok or not*.*” (Participant 1)*

Because of a lack of knowledge, many were not aware that cervical cancer was preventable, and some even believed there was no way to prevent it and this could lower their motivation to come for testing:

*“I believe anybody can get cervical cancer, unlike HIV which you can’t get if you protect yourself*.*” (Participant 1)*

Moreover, some of those who believed cervical cancer could be prevented did not seem to have the right ideas on how to prevent it. Of note, was a woman who associated it with diet and oily food:

*“By eating healthy. We have a problem of taking oily foods and foods that have been there for a long time, which is not good.” (Participant 3)*.

Almost all the women felt cervical smears were important, although many could not explain why. Many believed it was important simply because the healthcare workers said so and because one needs to know one’s status:

*“It’s important to get to know your status*. *You just have to know… Because that’s what the nurses always say …” (Participant 14)*

Some women anticipated that the test would be painful or that they would be embarrassed undressing in front of the nurses. Other women attributed new sensations in their body to the cervical smear procedure:

*“I believe pap test screening is important but there is something about pap smear screening, I do not know if it happens to all women or if it’s just me…before I did it I was just fine. Never had any problems but after doing it, I started having problems. It’s as if something is pulling me inside, especially when it [the weather] is cloudy …ever since I did it until now. I do not know if this was caused by pap smear or something else.” (Participant 12)*.

Others feared what the results might reveal and the possibility of a life threatening or incurable disease:

*“Some people when told that they are not well they give up on life. They think death is better*.*” (Participant 14)*

Not being aware that there is treatment for cervical cancer precursor lesions was another barrier. One woman even believed that whether or not you undergo any medical interventions, the outcome remains the same:

*“When it comes to cervical cancer, I have heard from people you might lose your life because whether or not you get help, it is still the same. You will die.” (Participant 1)*.

Some women believed that improved health education would increase their awareness and motivation to attend for cervical smears. They also commented that health education for HIV was much more intense and informative:

*“I can just say we need this educational forum so that we can understand. It is the same as when HIV started, there was little information given, but when they started laying it out then people started to understand.” (Participant 8)*.

Respondents also reported that alcohol misuse was common and impacted on people’s adherence to medical advice, including the need for cervical smears:

*“Where I stay, people take alcohol so even when advised to test they won’t even do it as they are always at the depot and everybody does what they want. They don’t really care about things, because they always drink alcohol…Even those who are on ARVS do not take them well. They go to the shebeen early in the morning and come back late and don’t take their medication and just throw the pills away. They drink too much and do not have time.” (Participant 9)*.

## Discussion

**Fig 1:**
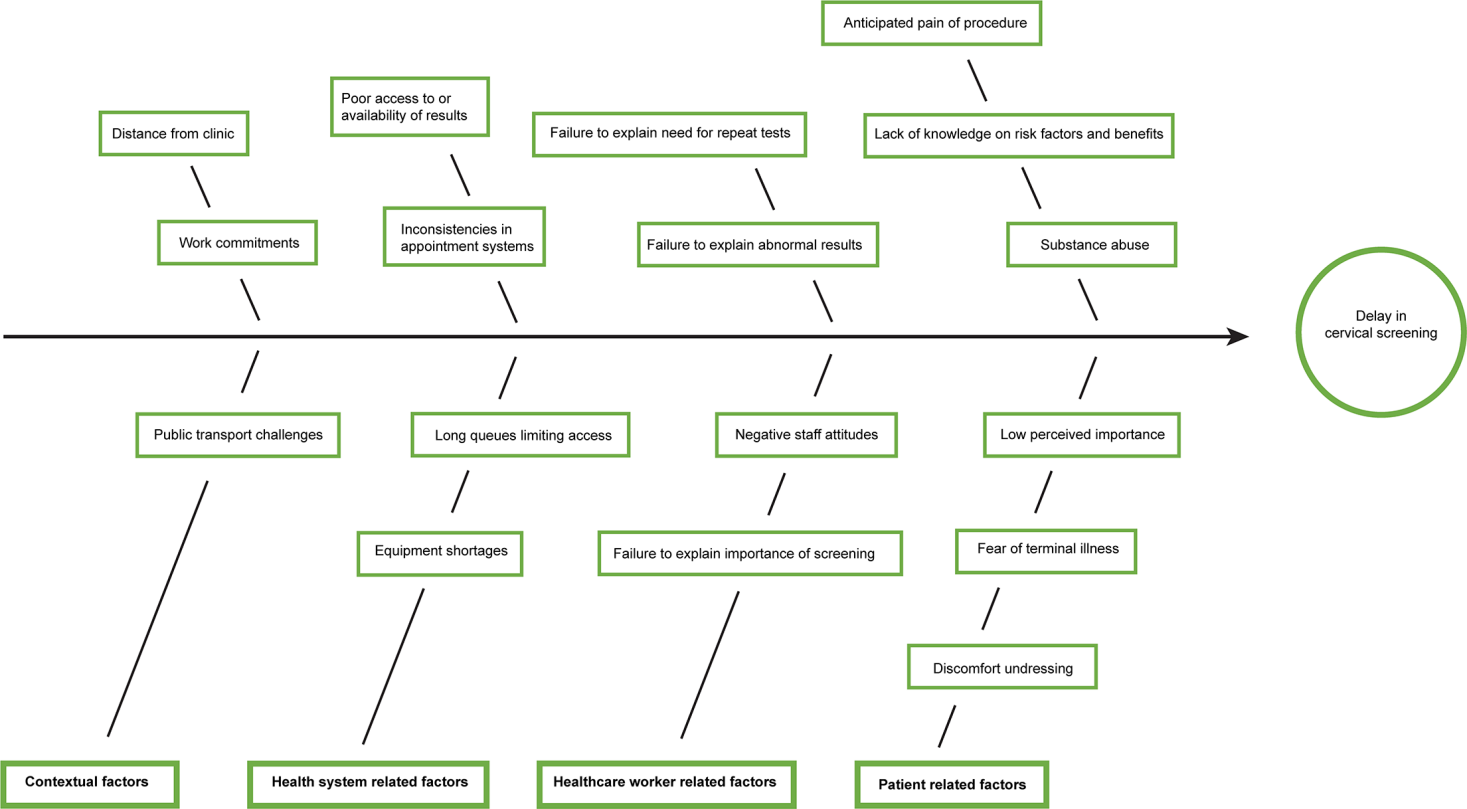
Fishbone diagram of factors that delay women from undergoing cervical cancer screening.

A wide range of factors delaying women from undergoing cervical cancer screening are summarised in Fig 1.

As noted elsewhere, many participants were not aware of the preventable nature of cervical cancer; the role of screening; the need for repeat cervical smears as well as the frequency of testing.[22] Similarly, they did not perceive themselves to be at an increased risk by virtue of their HIV status. They had concerns regarding the cervical smear procedure, such as embarrassment, discomfort, harm and violation of privacy.[15] Effective integration of health education on cervical cancer into services for HIV positive women has been associated with improved knowledge and understanding of risk in Uganda.[23]

A lack of patient-centred communication has been identified as a problem in primary care systems.[24] The effects of this on cervical screening are seen in terms of reduced acceptability of services and poor adherence to the screening process. Women may not understand the need for screening, the significance of abnormal results or the follow up expectations. Women specifically need to know why they should screen, when to start screening, how often to do it, what will happen if they do not participate and what abnormal results mean for them. Being more patient-centred, explaining procedures and their specific significance, adopting an attitude that encourages women to ask questions and maintaining good rapport are needed to improve utilisation of services. Healthcare workers also need to be conscious of myths and concerns, regardless of how trivial they may seem, such as a fear of undressing in front of male nurses or the anticipation of pain from speculum examination.

In many African district health services, the supply of equipment is problematic and staffing levels inadequate for optimal access to services.[25] The result of this is long queues and waiting times, which are frustrating to women and ultimately discourages them to undergo cervical cancer screening.[23] Adding to this frustration, is the failure to integrate health services, resulting in multiple queues where one could have sufficed. A further frustration is poor co-ordination of care with a failure to obtain results and poor continuity of care with a failure to implement an effective recall system.[23] The deficiencies in cervical cancer screening, therefore, are illustrative of the weaknesses in the primary care system as a whole in terms of supporting access, continuity of care, co-ordination of care and comprehensiveness.[26]

Inadequate health promotion has also been noted as a problem in regional primary care services and also applies to cervical cancer screening. In this particular study, many respondents highlighted the need for health education and health promotion to be intensified for cervical cancer as it is for HIV. The health belief model suggests that if women could perceive the importance of cervical screening then this might have a positive impact on the uptake of the service.[27] The health belief model also suggests that people’s beliefs about health problems and the perceived benefits of action and barriers to action, explain engagement (or lack of engagement) in health-promoting behaviour. A stimulus, or cue to action, such as health education, might also trigger health promoting behaviour.[27]

Alternative and emerging technology may help transcend the weaknesses in the primary care system identified in this study. Visual inspection tests with 3–5% acetic acid (VIA) and/or Lugol’s iodine (VILI) allows screening and treatment to be conducted at one visit without the need for laboratory services or recall. This has been shown to be effective in India and Sub-Saharan Africa and could be a better alternative to the traditional cervical smears.[25] Emerging technology includes rapid point of care human papilloma virus (HPV) tests that are performed in self-obtained vaginal samples. This may allow first line screening, triage of HPV-positive women and treatment.[25] This could also eliminate the need for multiple visits and laboratory tests.

The reliance on a single researcher had methodological strengths and weaknesses. On the one hand this ensured that interviews were conducted in a consistent manner, in the women’s home language and that translation was accurate. On the other hand the analytical process would have been stronger if the second researcher had performed a separate and independent analysis rather than just supervising the process. Unfortunately, the second researcher was not fluent in *Setswana*.

Other problems encountered were that the interviews tended to be slightly shorter than intended and many participants tended to give brief answers and had to be encouraged to elaborate further. Being interviewed by a medical doctor could have introduced an obsequiousness bias. The sampling included more people that had undergone cervical screening than had not and uneven numbers between age categories, which could potentially influence the range of themes identified. Findings were only derived from people in Kgatleng district and the reader must decide on how transferable the findings are to their own context.

Integrating cervical screening with visits for other problems, such as HIV management, would improve utilisation of cervical cancer screening. Otherwise attention should be given to an effective and consistent appointment system that allows women to attend at a convenient time and ensure sufficient resources are available. Telephonic communication should enable the scheduling and re-scheduling of appointments and text messaging to inform women about the availability of results or to recall them.

Health education and promotion should be intensified to raise awareness and understanding of cervical cancer screening using the general media, social media, community talks, clinic pamphlets or posters. Women’s education needs to be routinely incorporated into counselling for HIV as well as tailored in a patient-centred approach to individual women. Issues related to alcohol and substance abuse also need to be addressed through behaviour change counselling.

Introducing a VIA or HPV-based one-stop service for HIV-infected women may overcome many of the weaknesses in cervical cancer screening such as the problem of delayed results and treatment.

Further research could quantify the barriers identified in a descriptive survey with a representative sample of HIV positive women in order to measure how prevalent they actually are in the population.

## Conclusion

There is a significant deficit in knowledge of the benefits of cervical cancer screening amongst women living with HIV in Kgatleng district, Botswana. Although the participants heard that screening was important, they did not understand why and could not link it to prevention of cervical cancer. Participants had low levels of confidence with regard to the quality of the service due to poor patient-centeredness, inadequate appointment systems, unavailability of equipment and results. Moreover, there were many fears and misunderstandings regarding the screening process and interpretation of results.

Enhanced health promotion and education at both community and individual levels could help raise awareness around cervical screening and cancer. Strengthening the health system, particularly the appointment system and integration of different services, could improve utilisation of the service. Mitigating the weaknesses of the system through alternative technology that allows a one-stop test and treat service may also improve screening uptake.

## Supporting information

S1 File Interview guide. (DOCX)

## References

[1] Ibekwe C, Hoque M, Ntuli-Ngcobo B, Hoque M. Perceived barriers of cervical cancer screening among women attending Mahalapye district hospital. Botswana. Arch Clin Microbiol. 2011;2(1). doi: 10:3823/222

[2] Kawonga M, Fonn S. Achieving effective cervical screening coverage in South Africa through human resources and health systems development. Reprod Health Matters. 2008;16(32): 32–40. 10.1016/S0968-8080(08)32403-3 19027620

[3] Perng P, Perng W, Ngoma T, Kahesa C, Mwaiselage J, Merajver S, et al Promoters of Barriers to Cervical Cancer Screening in a Rural Setting in Tanzania. Int J Gynaecol Obstet. 2013;123(3): 221–225. 10.1016/j.ijgo.2013.05.026 24095307 PMC4291064

[4] Cain J, Denny L, Ngan H. Overcoming barriers to the eradication of cervical cancer: Women’s Health and Rights. Int J Gynaecol Obstet. 2007; 97(3): 232–234. 10.1016/j.ijgo.2007.03.004 17451715

[5] Victoria K, Mary S, Aaron J, Kevin A, Roger R. Barriers to cervical cancer screening in Mulanje, Malawi. Patient Prefer Adherence. 2011;5: 125–131. 10.2147/PPA.S17317 21448296 PMC3063659

[6] Ogedegbe G, Cassells A, Robinson C, DuHamel K, Tobin J, Sox C, et al Perceptions of barriers and facilitators of cancer early detection among low-income minority women in community health centers. J Natl Med Assoc. 2005;97(2): 162–170. 15712779 PMC2568778

[7] Fylan F. Screening for Cervical Cancer: A review of women’s attitudes, knowledge and behaviour. Br J Gen Pract.1998;48(433): 1509–1514 10024713 PMC1313202

[8] Daley E, Alio A, Anstey E, Chandler R, Dyer K, Helmy H. Examining barriers to cervical cancer screening and treatment in Florida through a socio-ecological lens. J Community Health. 2011;36(1): 121–131. 10.1007/s10900-010-9289-7 20559695

[9] Walling A. Social and Cultural Barriers to Cervical Cancer Screening. Am Fam Physician.2005;72(7): 1347.

[10] Fallala MS, Mash R. Cervical cancer screening: Safety, acceptability, and feasibility of a single-visit approach in Bulawayo, Zimbabwe. Afr J Prm Health Care Fam Med. 2015;7(1). 10.4102/phcfm.v7i1.742 26245601 PMC4564888

[11] United Nations International Children’s Emergency Fund. Botswana Country Profile. [cited 24 March 2017] Available from: http://www.unicef.org/infobycountry/Botswana_statistics.html#116

[12] Adjorlolo-Johnson G, Unger E, Boni-Ouattara E, Toure-Coulibaly K, Maurice C, Vernon S, et al Assessing the relationship between HIV infection and cervical cancer in Cote d’ Ivoire: A case-control study. BMC Infect Dis. 2010;10: 242 10.1186/1471-2334-10-242 20716343 PMC2933704

[13] Ministry of Health. Botswana National HIV/AIDS treatment guidelines. Gaborone: Ministry of Health, 2012.

[14] Markovic M, Kesic V, Topic L, Matejic B. Barriers to cervical cancer screening: a qualitative study with women in Serbia. Soc Sci Med. 2005;61(12): 2528–2535 10.1016/j.socscimed.2005.05.001 15953668

[15] Lim J, Ojo A. Barriers to utilisation of cervical cancer screening in Sub Saharan Africa: a systematic review. Eur J Cancer Care. 2017; 26(1). 10.1111/ecc.12444 26853214

[16] McFarland D: Cervical cancer and pap smear screening in Botswana: Knowledge and perceptions. Int Nurs Rev. 2003;50(3): 167–175 12930285 10.1046/j.1466-7657.2003.00195.x

[17] Ndejjo R, Mukama T, Musoke D. Uptake of Cervical Cancer Screening and Associated Factors among Women in Rural Uganda: A Cross Sectional Study. PLoS One. 2016;11(2): e0149696 10.1371/journal.pone.0149696 26894270 PMC4760951

[18] Idowu A, Olowookere S, Fagbemi A, Ogunlaja O. Determinants of Cervical Cancer Screening Uptake among Women in Ilorin, North Central Nigeria: A Community-Based Study. J Cancer Epidemiol. 2016 10.1155/2016/6469240 26880916 PMC4736774

[19] Mosavel M, Simon C, Oakar C, Meyer S: Cervical Cancer Attitudes and Beliefs-A Cape Town community. J Cancer Educ. 2009;24(2): 114–119 10.1080/08858190902854590 19431027 PMC3139476

[20] Reid S, Mash B. African Primary Care Research: Qualitative interviewing in primary care. Afr J Prm Health Care Fam Med. 2014;6(1). 10.4102/phcfm.v6i1.632 26245436 PMC4502895

[21] Mabuza LH, Govender I, Gihwala D, Ogunbanjo GA, Mash B. African Primary Care Research: Qualitative data analysis and writing results. Afr J Prm Health Care Fam Med. 2014;6(1). 10.4102/phcfm.v6i1.640 26245437 PMC4502868

[22] Cunningham M, Skrastins E, Fitzpatrick R, Jindal P, Oneko O, Yeates K, et al Cervical cancer screening and HPV vaccine acceptability among rural and urban women in Kilimanjaro Region, Tanzania. BMJ Open. 2015;5(3). 10.1136/bmjopen-2014-005828 25757944 PMC4360576

[23] Bukirwa A, Mutyoba J, Wanyenze R. Motivations and barriers to cervical screening among HIV infected women in HIV care: a qualitative study. BMC Womens Health. 2015;18: 82 10.1186/s12905-015-0243-9 PMC4603977 26458898

[24] World Health Organization. Primary Health Care: Now more than ever. Geneva: World Health Organization, 2008.

[25] Catarino R, Petignat P, Vassilakos P. Cervical cancer screening in developing countries at a crossroad: Emerging technologies and policy choices. World J Clin Oncol. 2015;6(6): 281–290. 10.5306/wjco.v6.i6.281 26677441 PMC4675913

[26] Kringos DS, Boerma WG, Hutchinson A, Van der Zee J, Groenewegen PP. The breadth of primary care: a systematic literature review of its core dimensions. BMC Health Serv Res. 2010;10(1):65 10.1186/1472-6963-10-65 20226084 PMC2848652

[27] Rosenstock Irwin M.; Strecher Victor J.; Becker Marshall H. Social learning theory and the health belief model. Health Educ Behav. 1988;15(2): 175–183. 10.1177/109019818801500203 3378902

