# Supplementary material for: Barriers to accessing cervical cancer screening among HIV positive women in Kgatleng district, Botswana: A qualitative study
Source: PLoS One. 2018 Oct 24;13(10):e0205425. doi: 10.1371/journal.pone.0205425 (PMC6200249; doi:10.1371/journal.pone.0205425)
Supplement: S1 File — (DOCX) [file pone.0205425.s001.docx]

Interview guide:

Interview questions:

This study wants to understand more about how women experience obtaining a pap smear at this clinic and whether there are any difficulties or barriers for them in doing this– can you tell me more about your experience of this in the clinic?

*Ideas and knowledge about cervical cancer screening:*

- What is your understanding of a pap smear?
- What do you think is meant to happen after you have a pap smear?
- If you have an abnormal pap smear what kind of treatment do you think is given?
- Who is at high risk of having cervical cancer?
- How preventable is cervical cancer?

*Importance of cervical cancer screening:*

- How important is it for you to get a pap smear at this clinic?
- How serious a disease do you think cervical cancer is?
- Have you ever had a pap smear?
- When was the last time you had a pap smear?
- How often do you go for a pap smear?

*Confidence in quality of service:*

- How confident are you in the quality of the service offered for pap smears?
- In your opinion, what would make it easier for women to go for pap smears?
- Are there any difficulties associated with accessing pap smears?
